# Impact of wind turbine sound on general health, sleep disturbance and annoyance of workers: a pilot- study in Manjil wind farm, Iran

**DOI:** 10.1186/s40201-015-0225-8

**Published:** 2015-10-12

**Authors:** Milad Abbasi, Mohammad Reza Monazzam, Arash Akbarzadeh, Seyyed Abolfazl Zakerian, Mohammad Hossein Ebrahimi

**Affiliations:** Department of Occupational Health Engineering, School of Public Health, Tehran University of Medical Sciences, Tehran, Iran; Department of Epidemiology and Biostatistics, School of Public Health, Tehran University of Medical Sciences, Tehran, Iran; Department of Occupational Health Engineering, School of Public Health, Shahroud University of Medical Sciences, Shahroud, Iran

**Keywords:** Noise annoyance, Sleep disturbance, General health, Wind turbine noise

## Abstract

**Background:**

The wind turbine’s sound seems to have a proportional effect on health of people living near to wind farms. This study aimed to investigate the effect of noise emitted from wind turbines on general health, sleep and annoyance among workers of manjil wind farm, Iran.

**Materials and methods:**

A total number of 53 workers took part in this study. Based on the type of job, they were categorized into three groups of maintenance, security and office staff. The persons’ exposure at each job-related group was measured by eight-hour equivalent sound level (LAeq, 8 h). A Noise annoyance scale, Epworth sleepiness scale and 28-item general health questionnaire was used for gathering data from workers. The data were analyzed through Multivariate Analysis of variance (MANOVA) test, Pillai’s Trace test, Paired comparisons analysis and Multivariate regression test were used in the R software.

**Results and discussion:**

The results showed that, response variables (annoyance, sleep disturbance and health) were significantly different between job groups. The results also indicated that sleep disturbance as well as noise exposure had a significant effect on general health. Noise annoyance and distance from wind turbines could significantly explain about 44.5 and 34.2 % of the variance in sleep disturbance and worker’s general health, respectively. General health was significantly different in different age groups while age had no significant impact on sleep disturbance. The results were reverse for distance because it had no significant impact on health, but sleep disturbance was significantly affected.

**Conclusions:**

We came to this conclusion that wind turbines noise can directly impact on annoyance, sleep and health. This type of energy generation can have potential health risks for wind farm workers. However, further research is needed to confirm the results of this study.

## Background

Wind energy compared to the other forms of traditional energy generation has fewer health effects and for this reason has positive health benefits [1]. However, compared with the health effects caused by unclean forms of traditional electricity generation, renewable energy generation is related to fewer adverse health effects [2]. Wind turbines generate noise that can be classified into a mechanical noise which is produced from the rotor or gearbox and an aerodynamic noise which is generated by turbulent wind flow near the wind turbine blades [3]. Wind turbine noise has remarkable audible Characteristics such as low frequency noise, amplitude modulation, impulsive and tonal Nature [4]. The adverse health effects of wind turbine noise on people can be categorized into the three groups such as Subjective effects (including annoyance, nuisance and dissatisfaction), Interference with activities (such as speech, sleep and learning) and Physiological effects (such as anxiety, tinnitus or hearing loss) [5]. One of the principal human responses to audible infrasound is an annoyance [5]. In the Pederson study (2007) except for annoyance, there was no direct adverse health effects associated with wind turbine noise. Pedersen showed that sleep problems and feelings of discomfort could be a secondary effect of the noise exposure that was related to noise annoyance [6]. Leventhall [7] concluded that wind turbine noise as a low frequency sound could have adverse effects on person’s health and cause sleep disorder. Noise annoyance can be one of the influencing factors for sleep disorder as they have reciprocal effects on sleep quality [8, 9]. According to the World Health Organization, noise annoyance has detrimental effects on health-related quality of life [10].

**Fig. 1:**
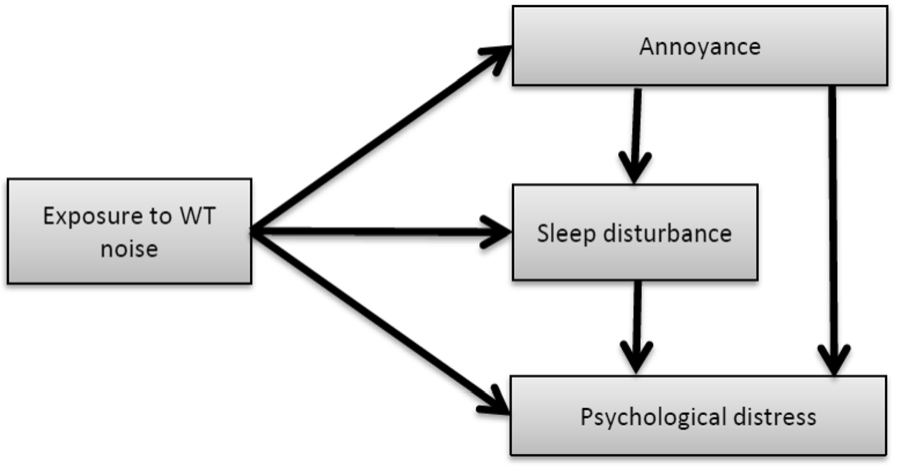
Structural model with noise exposure, annoyance, sleep disturbance and psychological distress

Wind Turbine Syndrome (WTS) is the clinical term for the collection of symptoms such as sleep disorders, headaches, tinnitus, nausea, irritability, loss of memory and concentration, nervousness, rapid heart rate, blood pressure, weight changes, abnormal heartbeat rhythms, mood problems, fatigue and depression experienced by many people living close to industrial wind turbines [11]. As a Multivariate approach, Bakker et al. [8] introduced a structural model that presented among exposure to wind turbine noise, psychological health, annoyance and sleep disturbance, as shown in Fig. 1.

Wind turbine noise can affect annoyance, sleep and health, and any disorder in these factors can amplify the sound exposure effects in the people living close to wind turbines. The effect of the noise exposure on the annoyance, sleep disturbance and health of the people living near to wind farms and their mutual effects on each other was studied in various places around the world and there is a comprehensive literature in this area [6–8], but, so far no study has been conducted to investigate these relations among wind farm workers. Due to the higher noise exposure in workers of wind power plant, as well as the long-term noise exposure, their health and sleep are roughly at the risk of adverse effects related to wind turbine noise. Thus, this study aimed to investigate the relation between exposure to the wind turbine noise and annoyance, sleep disturbance and general health of Manjil wind farm workers.

## Materials and methods

### Study area

This study was conducted in Manjil wind farm, Gilan, Iran and involved 53 voluntary workers of this wind power plant. Due to the mountainous topography and constant high wind speed in the Manjil district, this area is well-known for its wind energy utilization. This wind farm by 2009 used 171 NEG Micon and Vestas turbines with capacities ranging from 300 to 660 kW. The average wind speed in Manjil is approximately 14 m/s (at 40 m above ground).

### Population study

In this study, according to the distance from wind turbines and job similarities/differences, participants were categorized into three groups including; maintenance, security and administrative staffs. The maintenance workers repair and regulate wind turbines. In some cases, in the turbine trunk and in the vicinity of the turbine rotor, they are exposed to the extremely high level of noise.

The security staff has a further distance from the turbines compared to the maintenance workers. Official personnel are in an office building and due to farther distance from the turbines and insertion loss of barriers such as walls, windows, etc. they receives low level of noise compared to the other job groups.

### Sound measurement

The exposure level of employees at each job group to wind turbine sound was measured by eight-hour equivalent sound level (LAeq, 8 h) based on ISO 9612:2009 [12]. For this aim noise measurement was accomplished in 5, 5 and 2 locations for repairing, security, and official groups, respectively. For generalizing the results of exposure in various occupations, similarity of operations were considered. To achieve information about the features of sound generated by wind turbines, frequency analysis was performed in octave band using a calibrated sound level meter analyzer model TES 1358.

### Questionnaires

A questionnaire was designed to gather demographic data and employees’ background information such as age, Job tenure, Shiftwork type, education level, etc. moreover, a 28-item General Health Questionnaire (GHQ-28) was used to indicate psychological well-being and detect psychiatric morbidity. Information about Subjective noise annoyance and individuals’ sleep disorder were collected by noise annoyance scale and Epworth Sleepiness Scale (ESS), respectively.

The 28-item General Health Questionnaire (GHQ-28) has four sub-scales including: somatic symptoms, anxiety and insomnia, social dysfunction and depression. This questionnaire contains 24 questions with a three-point Likert scale (0 = Never, 3 = much more than usual). The total possible score on the GHQ 28 ranges from 0 to 84, where lower score indicates better psychological well-being. Reliability and validity of this questionnaire were approved by Goldberg [13]. Noise annoyance was determined based on the “Acoustics-Assessment of noise annoyance by means of social and socio-acoustic surveys” questionnaire which is provided in ISO/TS 15666 standard [14]. This scale included Likert items (0–10) with a high score indicating a high level of annoyance.

The EES contains 8 questions that ask people to rate, on a 4-point scale (0–3) their usual changes of dozing off or falling asleep in 8 different situations or activity. Most people engage in this 8 different situations or activity as part of their daily lives, although not necessarily every day. The total EES score is the sum of 8 items-scores and can range between 0 and 24. The higher the score, the higher the person’s level of daytime sleepiness would be. A number in the range of 10–24 is recognized abnormal (high sleepiness). The ESS has a global reliability and validity assessed by Cronbach’s alpha in the range of 73 to 88 % [15].

### Statistical analysis

In the last stage, the collected data were analyzed using R software. The Multivariate Analysis of variance (MANOVA) test was used to investigate significant differences in response variables such as sleep disturbance, annoyance and general health between noise exposure levels and various groups of age and work experience. The Pillai’s Trace test was done to examine the effects of research factors on response variables. The Paired comparisons of significant effects were conducted by Scheffe’s post hoc test. Multivariate regression test with Forward method was used to examine the influence of sleep disturbance, distance, noise annoyance, LAeq and age on the general health. Comparison of sleep disturbance and general health between different age groups, noise annoyance groups and various distances was done through MANOVA test.

## Results

Based on the results of this study, the values of 8 h equivalent noise level in A network for maintenance workers, security and office staff were equal to 83.66 and 60 dB, respectively. A total number of 53 employees of Manjil Wind Farm participated in this study. The mean (SD) age and work experience of the participants were obtained 30.8 (5.9) and 14.1 (5.5) years, respectively. Descriptive statistics of participants according to occupational groups are presented in Table 1.

**Table 1:** Descriptive statistics of participants according to occupational

	Total	Repairman	Security	Official staff
distance from wind (m)		0-50	50-100	≥150
Number of participants	53	22	17	14
Percent (%)	100	41.5	32	26.5
Mean(SD) of age (year)	38.1(5.9)	40.2(6.7)	37.5(3.8)	35.4(6)
Mean(SD) of experience (year)	14.1(5.5)	16.6(5.5)	13.2(3.8)	11.4(6.1)
…<diploma	12	4	6	2
diploma≤…	14	18	11	12

The average (SD) subjects’ general health, noise annoyance, sleep disturbance among all of the participants were obtained 23.6 (6.5), 6 (2.5) and 7.3 (3.1), respectively. As shown in Table 2, among all job groups, maintenance workers had more annoyance, sleep disturbance and general health. On other hand, in individuals with more than 19 years of job experience and individuals with more than 41 years of old had the least amount of noise annoyance, sleep disturbance and general health. Mean (SD) of subjects’ general health, noise annoyance, sleep disturbance according to demographic and background variables are presented in Table 2.

**Table 2:** General health, noise annoyance and sleep disturbance results in terms of job groups, age and experience

			General health	Noise annoyance	Sleep disturbance
Mean.(SD)	Total		23.6(6.5)	6(2.5)	7.3(3.1)
	Occupational group	Repairman	27.1(6.6)	8.4(1)	10.5(1.7)
		Security	22.3(4.3)	5.8(0.9)	6(1.4)
		Official staff	19.2(5.5)	2.6(1.3)	4(0.9)
	Age	…<36	21.6(5.3)	4.9(2.3)	6(2.4)
		36-41	22.3(5.1)	3.6(2.5)	7.5(3)
		41<…	30.2(7.3)	8.2(2)	10.2(3.2)
	Experience	…<12	19.6(4.5)	4.9(2.2)	5.6(2.4)
		12-19	24(5.1)	6.2(2.4)	7.4(2.8)
		19<…	29.9(7.3)	8.1(1.9)	10.1(3)

After a preliminary review of data, response variables between different age, work experience and noise exposure group there was no extreme outlier. On the other hand, review of the Minor outlier observations showed accuracy in data. The Multivariate Analysis Of Variance (MANOVA) test was used to investigate significant differences in response variables such as sleep disturbance, annoyance and general health between the three noise exposure levels and various groups of age and work experience. Bartlett’s Test of Sphericity revealed that there is a sufficient correlation between the dependent variables for performing the MANOVA (Chi-Square = 156. 26, *P* < 0.001). The hypothesis of equality of observed covariance matrices of the dependent variables, across groups was tested through Box’s M test. The test result indicated that, equality of covariance matrices of dependent variables was rejected among various levels of the independent variable. Thus, Pillai’s Trace test was used to study the effects of independent factors on general health, sleep and annoyance. Pillai’s Trace test results showed that, response variables were significantly different between three groups of noise exposure such that, noise exposure was able to explain 59 % of variances for response variables (Pillai’s F (6,90) =21.39, *P* < 0.001, Partial Eta^2^ = 0.59). Age had a significant multivariate effect on response variables and could explain about 19 % of the variance of the dependent variables (Pillai’s F (6, 90) =3.58, *P* = 0.003, Partial Eta^2^ = 0.19). Response variables had no significant difference between three groups of work experience (Pillai’s Trace = 0.16, NS, Partial Eta^2^ = 0.08). Assumption of equality of variances between all of response variables was proven by Levene’s test. Analysis of variance was performed to investigate the effect of noise exposure and age on three dependent variables separately. As shown in Table 3, noise annoyance was significantly different between three groups of noise exposure (F (2,46) = 113. 87, *P* < 0.001, Partial Eta^2^ = 0. 83). Noise exposure groups had a significant effect on sleep disturbance (F (2,46) = 85. 31, *P* < 0.001, Partial Eta^2^ = 0. 79). Finally, mean of general health among the three exposed groups was not equal and had significant difference between three groups of noise exposure (F (2, 46) = 4.69, *P* = 0.01, Partial Eta^2^ = 0.17). Moreover, noise annoyance was significantly different between age groups (F (2,46) = 5. 78, *P* < 0.01, Partial Eta^2^ = 0.2), But general health had no significant difference between different age groups (F (2,46) = 2.9, NS, Partial Eta^2^ = 0.11). Age had a significant effect on sleep disorders (F (2,46) = 5. 22, *P* < 0.01, Partial Eta2 = 0. 18). As shown in Table 3, According to the Adjusted R Squared, It is notable that, noise annoyance, sleep disturbance and general health was able to explain 86, 83.5 and 44.1 % of variations of model, respectively.

**Table 3:** Tests of between-subjects effects table

Source	Dependent variable	Type III sum of squares	Df	Mean square	F	Sig.	Partial Eta squared
Corrected model	Noise annoyance	303.023^a^	6	50.504	54.439	.000	.877
	Sleep disturbance	448.281^b^	6	74.714	44.986	.000	.854
	General health	1123.511^c^	6	187.252	7.838	.000	.506
Intercept	Noise annoyance	1496.806	1	1496.806	1613.444	.000	.972
	Sleep disturbance	2258.366	1	2258.366	1359.786	.000	.967
	General health	25150.229	1	25150.229	1052.750	.000	.958
LAeq	Noise annoyance	211.283	2	105.642	113.874	.000	.832
	Sleep disturbance	283.355	2	141.677	85.305	.000	.788
	General health	224.427	2	112.213	4.697	.014	.170
Age	Noise annoyance	10.732	2	5.366	5.784	.006	.201
	Sleep disturbance	17.339	2	8.670	5.220	.009	.185
	General health	140.418	2	70.209	2.939	.063	.113
Error	Noise annoyance	42.675	46	.928			
	Sleep disturbance	76.398	46	1.661			
	General health	1098.942	46	23.890			
Total	Noise annoyance	2302.000	53				
	Sleep disturbance	3424.000	53				
	General health	31798.000	53				
Corrected total	Noise annoyance	345.698	52				
	Sleep disturbance	524.679	52				
	General health	2222.453	52				

^a^R Squared = .877 (Adjusted R Squared = .860)

^b^R Squared = .854 (Adjusted R Squared = .835)

^c^R Squared = .506 (Adjusted R Squared = .441)

**Fig. 2:**
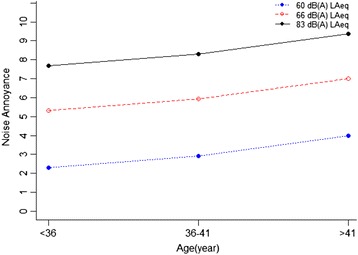
The relationship between amount of noise annoyance in age groups with different levels of noise exposure

**Fig. 3:**
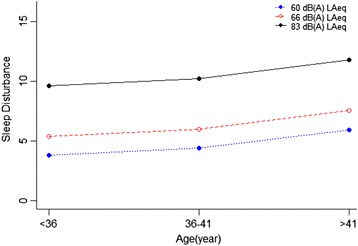
The relationship between amounts of sleep disturbance in age groups with different levels of noise exposure

**Fig. 4:**
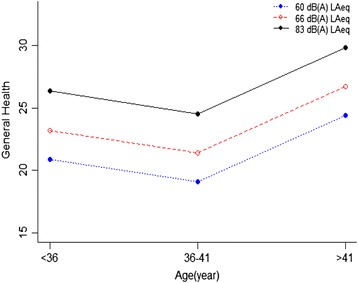
The relationship between amount of general health in age groups with different levels of noise exposure

Scheffe’s post hoc test was used to make pairwise comparisons of significant effects with bonferroni method. The pairwise comparison results revealed a significant difference in noise annoyance and sleep disturbance between all groups of age. As well as, pairwise comparisons of annoyance and sleep disorder showed significant differences between noise exposure groups, while, general health had significant differences only between two groups of noise exposure including 83 dB with 66 dB and 83 dB with 60 dB. These results are obvious in the estimated marginal means charts. As shown in Figs. 2, 3 and 4. The results of the estimated marginal means for each of the three response variables showed that as age and noise exposure increase then annoyance and sleep disturbance increase, as well as health effects decrease for participants that was between lower than 36 and 36–41 years old.

Multivariate regression analysis (due to forward method) was used to study the effects of sleep disturbance, distance from wind turbines, noise annoyance, noise exposure (LAeq, 8 h) and age on general health. The results showed that sleep disturbance and noise exposure had a significant effect on general health (F(2, 50) = 40.99, *P* < 0.001) and they can explain 61.2 % of changes in response variable. The effect of sleep disturbance and noise exposure on participants’ health was 2.62 and −0.39, respectively. The results of multivariate regression test have obtained in Table 4.

**Table 4:** Effects of sleep disturbance, distance from wind turbines, noise annoyance, noise exposure and age on general health

Dependent variables	Model compatibility		Model coefficients						
	F	P-value	B	Std. error	Beta	T	P-value	R	R^2^
Constant	40.99	0.001	32.4	6.34	-	*5.11	0.001	0.79	0.62
Sleep disturbance			2.62	0.39	1.27	*6.78	0.001		
LAeq			-0.39	0.12	-0.61	*-3.23	0.002		

*Significant at the 0.05 level

As well as Multivariate analysis of variance (MANOVA) was used to examine the influence of age, noise annoyance and distance from wind turbines on the sleep disturbance and general health. The result of Bartlett’s Test of Sphericity showed a significant Correlation between response variables (Chi-Square = 78.72, *P <* 0.001). on the other hand, Box’s M test results showed that, the hypothesis of equality of covariance matrices of dependent variables among various levels of the independent variables were established (Box’s M = 15.05, *P =* 0.2). Thus, Wilks’ Lambda test was carried out to study the effects of age, noise annoyance and distance from wind turbines on the sleep disturbance and general health. The result of Wilks’ Lambda test showed that noise annoyance and distance from wind turbines could significantly explain about 44.5 %,(F(18,76) = 3.39, *P <* 0.001, Partial Eta^2^ = 0.445) and 34.2 % of the variance in response variables, (F(4,76) = 9.86, *P <* 0.001, Partial Eta^2^ = 0.342), respectively. Age had a significant multivariate effect on response variables so that could explain approximately about 11.6 % of variance in the dependent variables (F(4,76) = 3.58, *P =* 0.04, Partial Eta^2^ = 0.116). Assumption of equality of variances in all response variables was proven by Levene test. The results of Between-Subjects analysis showed that noise annoyance had a significant effect on sleep disturbance (F (9,39) = 7.22, *P <* 0.001, Partial Eta^2^ = 0.625) and general health was significantly different between groups of noise annoyance (F(9,39) = 3.02, P = 0.008, Partial Eta^2^ = 0.41). The results of Analysis of variance for effect of age on two response variables showed that general health was significantly different in three age groups (F(2,39) = 3.44, P = 0.04, Partial Eta^2^ = 0.15) and age had no significant impact on sleep disturbance. The results were reverse for distance because it had no significant impact on health (F(2,39) = 1.27, NS, Partial Eta^2^ = 0.06), but sleep disturbance was significantly affected (F(2,39) = 23.9, *P <* 0.001, Partial Eta^2^ = 0.55). According to adjusted R squared, sleep disturbance and health explained, 92.7 and 55.7 % of Changes in Model, respectively. The results are shown in Table 5.

**Table 5:** Tests of between-subjects effects table

Source	Dependent variable	Type III sum of squares	Df	Mean square	F	Sig.	Partial Eta squared
Corrected model	General health	1484.529^a^	13	114.195	6.035	.000	.668
	Sleep disturbance	495.787^b^	13	38.137	51.480	.000	.945
Intercept	General health	19176.819	1	19176.819	1013.513	.000	.963
	Sleep disturbance	1693.626	1	1693.626	2286.150	.000	.983
Noise annoyance	General health	514.284	9	57.143	3.020	.008	.411
	Sleep disturbance	48.170	9	5.352	7.225	.000	.625
Age	General health	130.243	2	65.121	3.442	.042	.150
	Sleep disturbance	4.163	2	2.081	2.810	.072	.126
Distance	General health	48.213	2	24.106	1.274	.291	.061
	Sleep disturbance	35.373	2	17.687	23.874	.000	.550
Error	General health	737.924	39	18.921			
	Sleep disturbance	28.892	39	.741			
Total	General health	31798.000	53				
	Sleep disturbance	3424.000	53				
Corrected total	General health	2222.453	52				
	Sleep disturbance	524.679	52				

^a^R Squared = .668 (Adjusted R Squared = .557)

^b^R Squared = .945 (Adjusted R Squared = .927)

Scheffe’s post hoc test was used to do pairwise comparisons of significant effects. Based on health survey data, the group with the greatest noise annoyance showed a significant difference with other groups of noise annoyance. The result of sleep disorders was the same as health and generally, in all noise annoyance groups with more than 5 score there was a significant difference. General health had significant differences only between two groups of age, including age group with more than 41 years of old and group with less than 36 years old, as well as between group with more than 41 and group with 36–41 years of old. Finally, sleep disturbance among all of pairwise comparisons between groups of distance from wind turbine noise was statistically significant.

## Discussion

### Noise annoyance

The results of this study showed that exposure to noise from wind turbine had a statistically significant effect on annoyance of exposed workers and based on Adjusted R Squared, annoyance could justify 86 % of model Variations. Regardless of the type of noise source, this finding is in accordance with other study [16, 17]. Janssen et al. [18] declared that noise from wind turbines is more annoying than environmental sources such as road and rail traffic noise. One of the characteristics of wind turbine noise like low frequency nature is the reason that wind turbine noise can cause annoyance [19]. In the present study, there was a linear relation between noise exposure and noise annoyance, and workers who had higher noise exposure experienced higher level of annoyance. This result is consistent with Bakker et al. [8] study, which was accomplished among people who lived near to wind farms. They reported that the level of annoyance depended on the level of their noise exposure, a higher exposure increased the possibility of being annoyed [6, 8]. The visibility of turbines had negative impact on the annoyance of residents [20] and in this study office staff that can’t see wind turbine, had lower noise annoyance. Shadow flicker is another feature of wind turbine that can cause annoyance in people. Noise exposure and age have an additive effect on annoyance of workers and a simultaneous increase in these two variables can dramatically increase the level of annoyance.

The results showed a significant positive correlation between the noise annoyance and worker’s age and the higher the age, the higher noise annoyance. Hearing loss at high frequencies because of aging process can removes the coating effect of background noise to the wind turbines noise [21]. Thus, it can be supposed that older people experience high level of low frequencies noise due to the decrease in the coating effect for background noise, the older people are more sensitive to low frequency noise generated from wind turbines that make them annoyed [22]. Noise sensitivity and economic benefit also have significant impact on annoyance of people living near to wind farm [8, 23]. Annoyance among people who are more sensitive to noise is higher than insensible people. Workers are considered as economically beneficiaries of wind power plants and previous studies confirmed that beneficiaries have been less annoyed than other [18]. Due to the higher level of workers noise exposure than residents living near to wind farm, we expected the workers to be more annoyed than general population, but our expectation did not come true which could be because of workers economic benefits from wind power plant, as well as, may be explained by the fact that general populations are more sensitive than workers.

### Sleep disturbance

The results of present study showed that, sleep disturbance between noise exposure groups (job groups) and groups with different age had a statistically significant difference. Results were in accordance with Kim et al. [24] study that showed a dose–response relationship between the airplane sound level and sleep quality. In another study (2013) on people living close to wind turbines, a significant association was reported between the distance from turbines and sleep disturbance [25]. Bruck and his colleague (2009) found that low frequency noise was more effective for arising people from deep sleep [26]. In the previous literature, age was introduced as a predictor for sleep [27, 28]. Solet et al. [29] said that impulsive noise causes more sleep disorder than other types of sound. Solet and his colleagues verify the finding of the present study because wind turbine noise has an impulsive nature. Despite, more noise exposure of workers than the general population, sleep disturbance in the current study is less than that reported by people living near to wind farms. It could be due to hiding the truth about their sleep disorders because of fear of punishment by their superiors, Loss of financial interests due to job loss, more knowledge, strength and consistency of the workers compared to the ordinary people. Nissenbaum et al. [30] showed an exposure-response association between the distances from wind turbines and sleep disorder within the distance of 1.4 km from the industrial turbines. In the near distance from the noise source, people received more noise and dramatically response to that. Contrary to the obvious differences in methods, tools, and materials used, the results of Nissenbaum and the present study both confirmed the dose–response relationship between wind turbine sound exposure and sleep disorder. Akerstedt et al. [31] said that age can cause poorer sleep quality. Dijk and Duffy [32] studied changes in the circadian during the aging process. They declare that changes in the sleep process such as drops in slow-wave sleep and sleep circles, as well as, a reduced strength of the circadian signal promoting sleep in the early morning hours can be associated with age-related changes in the circadian. Sedative drugs use, having sickness and having a second job can strongly interfere with workers sleep that was not within the scope of the present study.

### General health

The results of this study showed that exposure to the noise generated by wind turbine had a significant effect on general health. A significant difference in general health score among various noise exposure levels only was between 83 dB with 66 dB and 83 dB with 60 dB. It can be stated that, exposure to higher levels of noise, can cause more adverse health effects. The turbine vibration is a risk factor that amplifies the sound effects on maintenance workers. As well as, the difference in salary could be one of the roots of differences in perceived effects.

Health decreased in 36–41 years of age could be due to Compatibility with work conditions in this age range. The analysis results showed that age-increasing alone had no effect on health, but, health been adversely affected by simultaneously increasing age and noise exposure. Based on these results, It can be concluded that age strengthen the effect of noise exposure on general health. Krogh et al. study (2011) reported that people living near wind turbines had various health complaints [33], as well as Nissenbaum and his coworkers said that wind turbine noise at different distance had an different severity of adverse effect on the health. Many studies have been done to investigate the impact of noise generated by wind turbine on the health, but there is no clear understanding of the mechanisms of this effect. Several assumed mechanisms are included as nervousness, exposure to low frequency noise, visual impact, noise sensitivity, noise annoyance, sleep disorder, attitude to the sound sources and individual characteristics [34–37]. Noise is clearly identified as a factor of stress and stress may be considered as the possible mechanism through which mental and physical health can be affected by noise [38].

Multivariate regression test results showed that sleep disturbance and noise exposure had a significant effect on general health and they can explain 61.2 % of changes in response variable. The effect of sleep disturbance and noise exposure on participants’ health was 2.62 and −0.39, respectively. Based on the result, we can see that sleep disturbance compared to noise exposure has greater effect on health. Thus, Noise can disturb sleep of exposed people and in this way, effect on their health. Sleep disorders can cause anxiety that is a reason for other adverse health effects in people who live near wind farms [35]. Leventhall [7] said that wind turbine sound may have serious effects on individuals’ health and cause sleep disorder. Baker showed a relationship between sleep disturbance and psychological distress. Thus, the wind turbine can effect on health through direct (by generated noise) and indirect (by caused sleep disturbance) mechanism. As well as Multivariate analysis of variance (MANOVA) was used to examine the influence of age, noise annoyance and distance from wind turbines on the sleep disturbance and general health. the result of analysis test showed that noise annoyance had a significant effect on sleep disturbance and general health. The results of Analysis of variance for effect of distance on two response variables showed that distance from wind turbine noise had no significant impact on health, but sleep disturbance was significantly affected by distance. Noise annoyance cause from anxiety and conflict and if it continues to increase, could cause deterioration of the health and welfare [23]. Dratva et al. [39] said that noise annoyance was associated with health related quality of life. It can be claimed that the effect of noise exposure may be mediated through annoyance rather than through a direct exposure effect [39]. Bakker showed that annoyance can lead to sleep disturbance and psychological distress and there was no direct relation between exposure to the sound of wind turbines and sleep disturbance or psychological distress. Annoyance can be considered as a mediator between sound exposure and sleep disturbance, and also between sound exposure and psychological distress [8]. The previous studies reported that there was an interaction between annoyance and psychological distress as well as between annoyance and sleep [9, 40]. According to the result of this study, distance from wind turbine had no effect on the psychological distress but significantly affect sleep disturbance. It can be said that due to less distance from wind turbines, maintenance workers declare more sleep disturbance that cause more psychological distress.

## Conclusion

We concluded that wind turbine noise can directly impact on annoyance, sleep and health. The severity of the effects of noise on each of these indicators of health is related to general health conditions of people. The poorer health led to the more effects of noise generated by wind turbine on the annoyance, sleep and psychological distress. It is suggested that, pre- employment health assessment must be done for new recruits to eliminate any reinforcing factors for effects of exposure to sound from wind turbine.

## Suggestions for further research

The authors recommend that, future studies in wind power plants, study the effect of wind turbine noise on health, relying on risk factors such as noise sensitivity, Sedative drugs use, visual impact, shadow flicker effects, shift work and job stress.
